# Development and partial validation of an RT-qPCR assay for the rapid detection of spring viremia of carp virus (SVCV)

**DOI:** 10.3389/fmicb.2025.1726705

**Published:** 2026-01-23

**Authors:** Peng Zhu, Jie Sun, Lishan Liao, Zhiheng Zuo, Annabel Rice, Shishun Gui, Jiang Wu, Yumin Zhu, Lei Zhang, Hongwei Liu, David Stone, Hong Liu

**Affiliations:** 1 Animal and Plant Inspection and Quarantine Technology Centre, Shenzhen Customs, Shenzhen, China; 2 College of Life Sciences and Oceanography, Shenzhen University, Shenzhen, China; 3 College of Fisheries and Life Sciences, Shanghai Ocean University, Shanghai, China; 4 College of Life Science and Technology, Jinan University, Guangzhou, China; 5 Center for Environment, Fisheries and Aquaculture Science, Weymouth, United Kingdom; 6 Comprehensive Technical Service Center of Dandong Customs, Dandong, Liaoning, China

**Keywords:** diagnostic assay, fish disease surveillance, RT-qPCR, spring viremia of carp virus, viral detection, WOAH validation

## Abstract

Spring viremia of carp (SVC), caused by spring viremia of carp virus (SVCV), is a highly contagious disease that poses a serious threat to cyprinid aquaculture and international trade, and it is listed as a notifiable disease by the World Organization for Animal Health (WOAH). Effective surveillance and control of SVCV rely on accurate and highly sensitive molecular diagnostic methods. However, several previously published RT–qPCR assays contain mismatches between primer/probe sequences and viral genomes, which may lead to false-negative results and reduced diagnostic reliability. In this study, a whole-genome comparison of 24 representative SVCV strains covering all four genotypes (SVCVa–d) was conducted, and a new primer–probe set (Cefas AR) targeting a highly conserved region of the L gene was designed. Reaction conditions were optimized, and the assay was rigorously validated in accordance with the WOAH Manual of Diagnostic Tests for Aquatic Animals. The developed RT–qPCR assay exhibited excellent analytical performance, with a limit of detection of 1.28 copies/μL, diagnostic sensitivities of 100% for cell-culture isolates and 96.6% for tissue samples, and a diagnostic specificity of 100%. In addition, the assay demonstrated strong reproducibility and consistency across nine independent laboratories. In conclusion, the WOAH-validated RT–qPCR assay developed in this study provides a highly sensitive, specific, and reliable tool for rapid screening, routine surveillance, and confirmatory diagnosis of SVCV, supporting sustainable aquaculture development and international aquatic animal health management.

## 1 Introduction

Spring viremia of carp (SVC) is an acute, systemic disease of cyprinid fishes caused by the spring viremia of carp virus (SVCV). SVCV primarily infects freshwater fishes of the family *Cyprinidae*, posing substantial risks to the ornamental fish trade, recreational angling, and aquaculture-based food production (Ashraf et al., 2016; Walker et al., 2022). The common carp (*Cyprinus carpio*) is considered the principal host; however, natural outbreaks have also been reported in multiple species, including koi carp (*C. carpio koi*), goldfish (*Carassius auratus*), crucian carp (*C. carassius*), grass carp (*Ctenopharyngodon idella*), and bighead carp (*Aristichthys nobilis*) (Tesarcík et al., 1977; Ahne et al., 2002; Sanders et al., 2003; Ashraf et al., 2016). It was traditionally considered a disease confined to Europe for decades, but it has since been detected in Asia and appears to have spread to North America (Miller et al., 2007; Soliman et al., 2008; Shao and Zhao, 2016). Because of its substantial economic impact and risk of transboundary spread, SVC is listed by the World Organization for Animal Health (WOAH) as a notifiable disease. Early and reliable detection is therefore critical for surveillance, movement control, and outbreak management in carp aquaculture (Zhang et al., 2024).

SVCV belongs to the family *Rhabdoviridae* and the genus *Sprivivirus* (Walker et al., 2022). It exhibits the typical bullet-shaped morphology of vertebrate rhabdoviruses, measuring 80–180 nm in length and 60–90 nm in diameter (Tesarcík et al., 1977). It is a negative-sense single-stranded RNA virus with a genome of approximately 11 kb, encoding five proteins: nucleoprotein (N), phosphoprotein (P), matrix protein (M), glycoprotein (G), and RNA-dependent RNA polymerase (L) (Teng et al., 2007; Wang et al., 2020). Based on variations in the G gene, SVCV is classified into four genotypes: SVCVa, SVCVb, SVCVc, and SVCVd (Stone et al., 2003).

The traditional method for SVCV detection typically involves virus isolation on fish cell lines, such as the fathead minnow cell line (FHM), the Epithelioma papulosum cyprini cell line (EPC), and the grass carp ovary cell line (GCO), performed at 15 °C (Wang et al., 2016; Jiao et al., 2023; International Office of Epizootics, Aquatic Animal Health Standards Commission, 2024). The isolated material is then confirmed by reverse transcription polymerase chain reaction (RT-PCR) and sequencing, which is considered the “gold standard” for SVCV detection (Koutná et al., 2003; Stone et al., 2003). In addition, there are antibody-based methods such as the neutralization test (NT), the indirect fluorescent antibody test (IFAT), and the enzyme-linked immunosorbent assay (ELISA) (Faisal and Ahne, 1984; Way, 1991). However, because SVCV shares cross-reactive antigens with closely related rhabdoviruses, such as pike fry rhabdovirus (PFRV), these immunological assays can only provide a presumptive diagnosis of SVCV (Vicenova et al., 2011).

At present, nucleic acid–based assays have been widely applied for pathogen detection and identification. Several nucleic acid detection methods have been developed for SVCV, including reverse transcription polymerase chain reaction (RT-PCR) (Stone et al., 2003; Shimahara et al., 2016), reverse transcriptase quantitative PCR (RT-qPCR) (Yue et al., 2008), and loop-mediated isothermal amplification (LAMP) (Shivappa et al., 2008). However, except for RT-PCR, these existing methods have not undergone systematic performance evaluation in accordance with the WOAH validation framework—covering Stage 1 (analytical performance), Stage 2 (diagnostic performance), Stage 3 (reproducibility), and Stage 4 (implementation). Therefore, these nucleic acid–based methods have not yet been formally recommended by WOAH (WOAH, 2023). Although WOAH recommends conventional RT-PCR as the confirmatory diagnostic method for SVCV, its reliance on electrophoresis and sequencing, relatively long turnaround time, and limited suitability for high-throughput testing underscore the need to establish a more rapid, quantitative, and diagnostically robust RT-qPCR assay.

Currently, 20 complete SVCV genome sequences have been published. Among these, the Björklund strain (NC_002803) belongs to genogroup SVCVd (Björklund et al., 1996; Hoffmann et al., 2002), whereas the majority of the remaining sequences fall within genogroup SVCVa, including SVCV_2022 (OP495631), SVCV-265 (KJ513477), A1 (DQ097384), A2 (DQ491000), SH140501 (KR012465), SH140502 (KR012466), SH140503 (KR012467), SH140522 (KR012468), BJ0505-2 (EU177782), shlj4 (MT675953.1), 2016 (PQ807631.1), SH160901 (KY475636), ADC-SVC2016-1/3/5 (MG663514/MG663513/MG663512), as well as KT321307, AJ318079, MZ343157, and KU230365 (Teng et al., 2007; Zhang et al., 2009; Xiao et al., 2014; Kim et al., 2016). Previously published RT-qPCR assays were developed when only a limited number of complete genome sequences were available, and therefore were primarily designed based on a narrow sequence dataset (Rice, 2022). Our analyses revealed sequence mismatches between the primer/probe sets used in these assays and the genomes of various circulating SVCV strains, which may compromise analytical sensitivity and adversely affect diagnostic accuracy. Here, we systematically analyzed 24 representative SVCV strains across all four genotypes to redesign new specific primers and probes (Cefas AR) based on the conserved regions of the L gene. The reaction system and detection conditions were subsequently optimized. Finally, the established assay was comprehensively validated in accordance with the principles and procedures outlined the WOAH Manual of Diagnostic Tests for Aquatic Animals (WOAH, 2023).

## 2 Materials and methods

### 2.1 RT-qPCR primer and probe design

**FIGURE 1:**
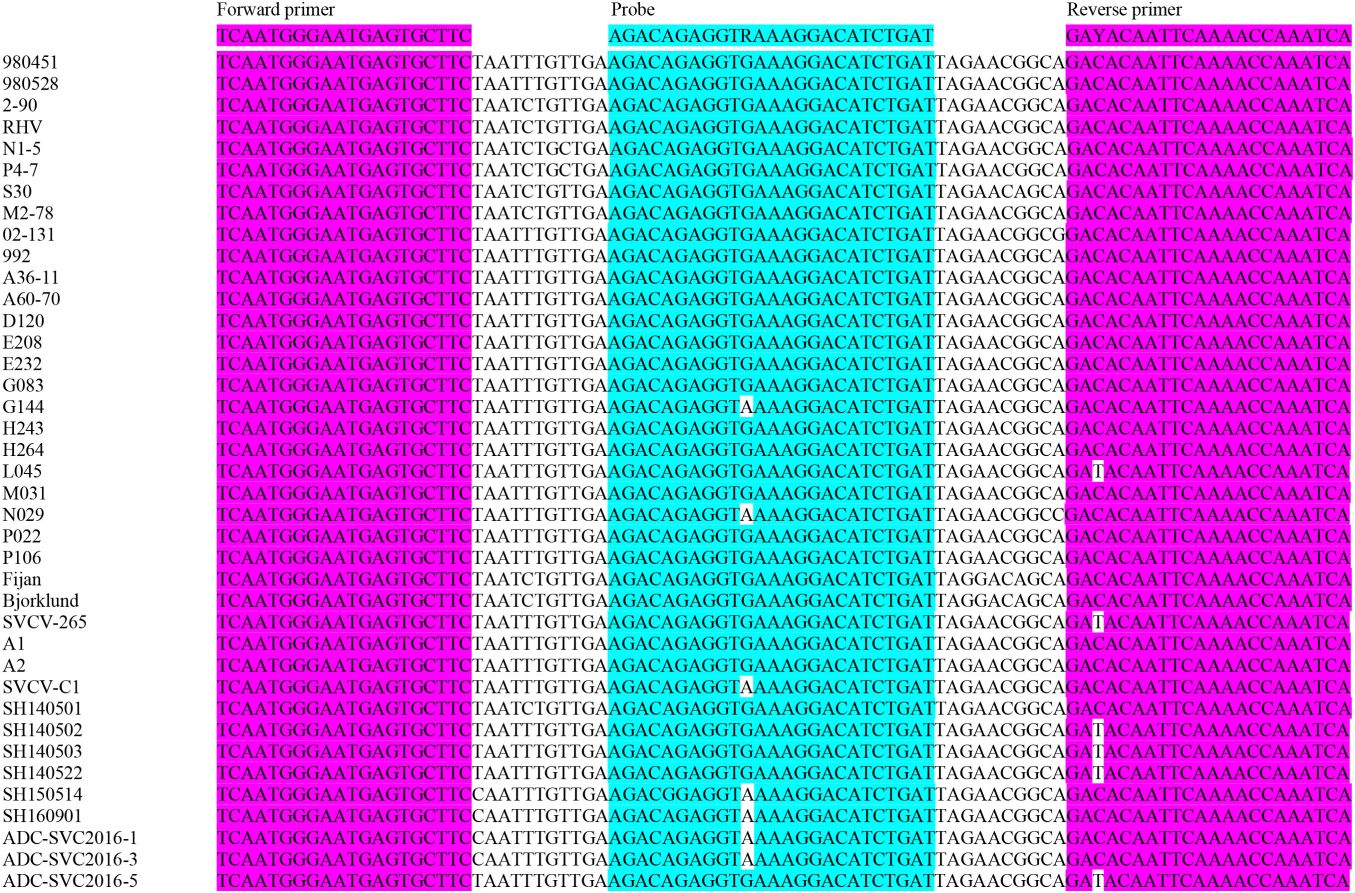
The SVCV L gene region selected for the TaqMan primer and probe design.

To ensure inclusivity across all four SVCV genotypes, we compiled 39 genomes from NCBI GenBank and our Illumina sequencing (SVCVa, *n* = 31; SVCVb, *n* = 2; SVCVc, *n* = 2; SVCVd, *n* = 2) (Supplementary Table 1). Multiple sequence alignment was performed with MAFFT (v7) to identify highly conserved regions within the L gene, on the basis of which TaqMan primers and probe were designed using Primer3 (Figure 1). The primer pair yields an 87-bp amplicon; the probe carries a FAM reporter at the 5′ end and a BHQ1 quencher at the 3′ end (Table 1).

**TABLE 1:** Primer and TaqMan probe sequences used in this study for RT-qPCR.

Primer/Probe	sequence(5′–3′)
SVCV Cefas AR-F	5′-CAATGGGAATGAGTGCTTCT-3′
SVCV Cefas AR-R	5′-TGATTTGGTTTTGAATTGTRTC-3′
SVCV Cefas AR-P	5′-FAM-AGACAGAGGTRAAAGGACATCTGAT-BHQ1-3′

### 2.2 Optimization of reaction conditions

RT-qPCR was used the AgPath-ID One-Step RT-qPCR Kit (Thermo, United States), following the manufacturers protocol. The total volume of the reaction system was 25 μl, containing 12.5 μL of 2X RT-PCR Buffer, 1 μL (10 μM) of Forward and reverse PCR primers, 0.5 μL (10 μM) of TaqMan probes, 1 μL of 25X RT-PCR Enzyme Mix, 5 μL of RNA template and 4 μL of ddH_2_O. Temperature cycling conditions consisted of a reverse transcription step at 45°C for 15 min, followed by 95°C for 10 min and 40x cycles of 95°C for 15 s and 55–60°C for 1 min.

To optimize the annealing temperature of the RT-qPCR assay, viral RNA extracted from eight SVCV strains representing four genotypes (SVCVa, SVCVb, SVCVc, and SVCVd) was used (Table 2). Gradient RT-qPCR was performed under the reaction conditions described above, with annealing temperatures ranging from 55 to 60°C (1°C increments), to identify the optimal temperature that ensured efficient amplification across all genotypes.

**TABLE 2:** Collection of viral strains of different host origin and differing genotypes used for evaluation of RT-qPCR.

No.	Subtype	Host	Origin
20030,772	SVCVa	*Cyprinus carpio*	Shanghai, China
20040741	SVCVa	*Cyprinus carpio*	Jiangsu, China
20070520	SVCVa	*Cyprinus carpio*	Heilongjiang, China
20080136	SVCVa	*Cyprinus carpio*	Jiangsu, China
20120444	SVCVa	*Cyprinus carpio*	Henan, China
RHV	SVCVb	*Oncorhynchus mykiss*	Ukraine
N1-5	SVCVc	*Aristichthys nobilis*	Ukraine
10/3	SVCVd	unknown	United States of America

### 2.3 Analytical specificity

Nine non-target viruses (Table 3) and muscle tissues from 8 species of fish (including *Ctenopharyngodon Idella*, *Carassius carassius*, *Cyprinus carpio*, *Hypophthalmichthys molitrix*, *Aristichthys nobilis*, *Mylopharyngodon piceus*, *Danio rerio*, and *Siniperca chuatsi*), all confirmed to be healthy and free of other pathogen infections, were used to evaluate the analytical specificity of the established RT-qPCR method.

**TABLE 3:** Viruses information for Analytical specificity test.

No.	Name	Host	Origin
ZJLYRV20309	Largemouth bass rhabdovirus, MSRV	*Micropterus salmoides*	Zhejiang, China
ZJLYRV19830	Largemouth bass ranavirus, LMBV	*Micropterus salmoides*	Zhejiang, China
Bvh1 VR890	Infectious pancreatic necrosis virus, IPNV	unknown	ATCC
20161690	Infectious haematopoietic necrosis virus, IHNV	*Oncorhynchus mykiss*	Sichuan, China
20160839	Grass carp hemorrhage virus, GCHV	*Ctenopharyngodon Idella*	Guangxi, China
20142788	Viral hemorrhagic septicemia virus, VHSV	unkown	Fish disease Proficiency Test
20141224	Goldfish haematopoietic necrosis virus, GFHNV	*Carassius carassius*	Jiangsu, China
2008–0113	Hirame rhabdovirus, HRV	*Paralichthys dentatus*	Shandong, China
2006	Koi herpesvirus, KHV	*Cyprinus carpio*	Guangxi, China

### 2.4 Analytical sensitivity

The target fragment for the TaqMan RT-qPCR assay was synthesized based on the sequencing data by Sangon Biotech (Shanghai) Co., Ltd., and subsequently cloned into the pUC57 plasmid (designated pUC57-SVCV). This recombinant plasmid, containing the target sequence, served as the DNA standard. The copy number of the recombinant plasmid was calculated as 1.28 × 10^8^ copies/μL according to the following formula. A 10-fold serial dilution of the pUC57-SVCV plasmid was prepared and tested in triplicate at each dilution level to evaluate the analytical sensitivity of the assay.


$$\mathrm{Copies}/\mathrm{ul}=\frac{6.02\times 10^{23}\times 10^{-9}}{\mathrm{DNA}\,\mathrm{length}\times 660}$$

### 2.5 Assay repeatability

To test whether the SVCV RT-qPCR assay is repeatable, three different concentrations of SVCV standard plasmids (pUC57-SVCV) were tested. For each concentration, the reactions were performed in technical triplicates using the same machine and the same operator. The experiment evaluated the standard deviation (SD) and coefficient of variation (CV) of the three results at each concentration to test the repeatability of the assay.

### 2.6 Diagnostic sensitivity and diagnostic specificity test

Diagnostic sensitivity (DSe) and diagnostic specificity (DSp) are key performance indicators for assay validation. In this study, a validation panel was used to evaluate the performance of the established RT-qPCR assay. The validation panel consisted of 264 cell-culture isolates and 204 tissue samples (Table 4). The 264 cell-culture isolates were derived from original diseased fish samples collected between 2002 and 2022 from various host species, including common carp (*Cyprinus carpio*), goldfish (*Carassius auratus*), crucian carp (*C. carassius*), grass carp (*Ctenopharyngodon idella*), koi carp (*C. carpio var. koi*), Chinese sturgeon (*Acipenser sinensis*), and other species. The diseased fish tissue samples were processed following the WOAH-recommended virus isolation and semi-nested RT-PCR methods for virus separation and identification. The resulting cell-culture isolates were subsequently stored at −80 °C in the SVCV reference laboratory. Of the 204 tissue samples, 189 tissue samples were obtained from experimentally infected koi carp, in which four SVCV strains (10-3, 20040741, 2022-SC, and 20120450) were used for infection. The remaining 15 tissue samples were collected from aquaculture farms in Shandong province, Inner Mongolia Autonomous Region (Neimeng) and Tianjin to further assess the practical applicability of the assay. Immediately after sampling, all tissues were placed in RNase-free microcentrifuge tubes, stored at low temperature, and promptly processed for nucleic acid extraction using the MagMAX-96 Viral Isolation Kit (Thermo Fisher Scientific) to ensure RNA integrity. The diagnostic sensitivity and specificity were calculated according to the formulas DSe = TP/(TP + FN) and DSp = TN/(TN + FP).

**TABLE 4:** Summary of the validation panel used to evaluate the diagnostic performance of the RT-qPCR assay.

Category		Subtyp/Strain	Number of samples	Host species	Year of collection
Cell culture isolates		SVCVa	180	Common carp	2002–2022
		SVCVa	25	goldfish	
		SVCVa	8	crucian carp	
		SVCVa	6	grass carp	
		SVCVa	25	Koi carp	
		SVCVa	1	Chinese sturgeon	
		SVCVa	11	others	
		SVCVb	2	others	
		SVCVc	3	others	
		SVCVd	3	others	
Tissue samples	Experimental infection	20040741	63	Koi carp	2022 2024
		10–3	63	Koi carp	
		20100910	63	Koi carp	
	Field samples	None	15	Common carp	

### 2.7 Assay reproducibility

To evaluate the reproducibility of the method, five blinded samples were selected, comprising a strong-positive sample (Ct 20–25), a moderate-positive sample (Ct 25–31), a weak-positive sample (Ct 31–35), and two negative samples. Each sample was subdivided into nine aliquots and distributed to nine laboratories. All laboratories tested the aliquots using a uniform primer–probe set supplied by our laboratory; however, the nucleic acid extraction kits and RT-qPCR reagents were not strictly standardized across sites. Results from all laboratories were then collated and analyzed to assess the assay’s stability and reproducibility under varying experimental conditions.

## 3 Results

### 3.1 Optimal of reaction conditions

**FIGURE 2:**
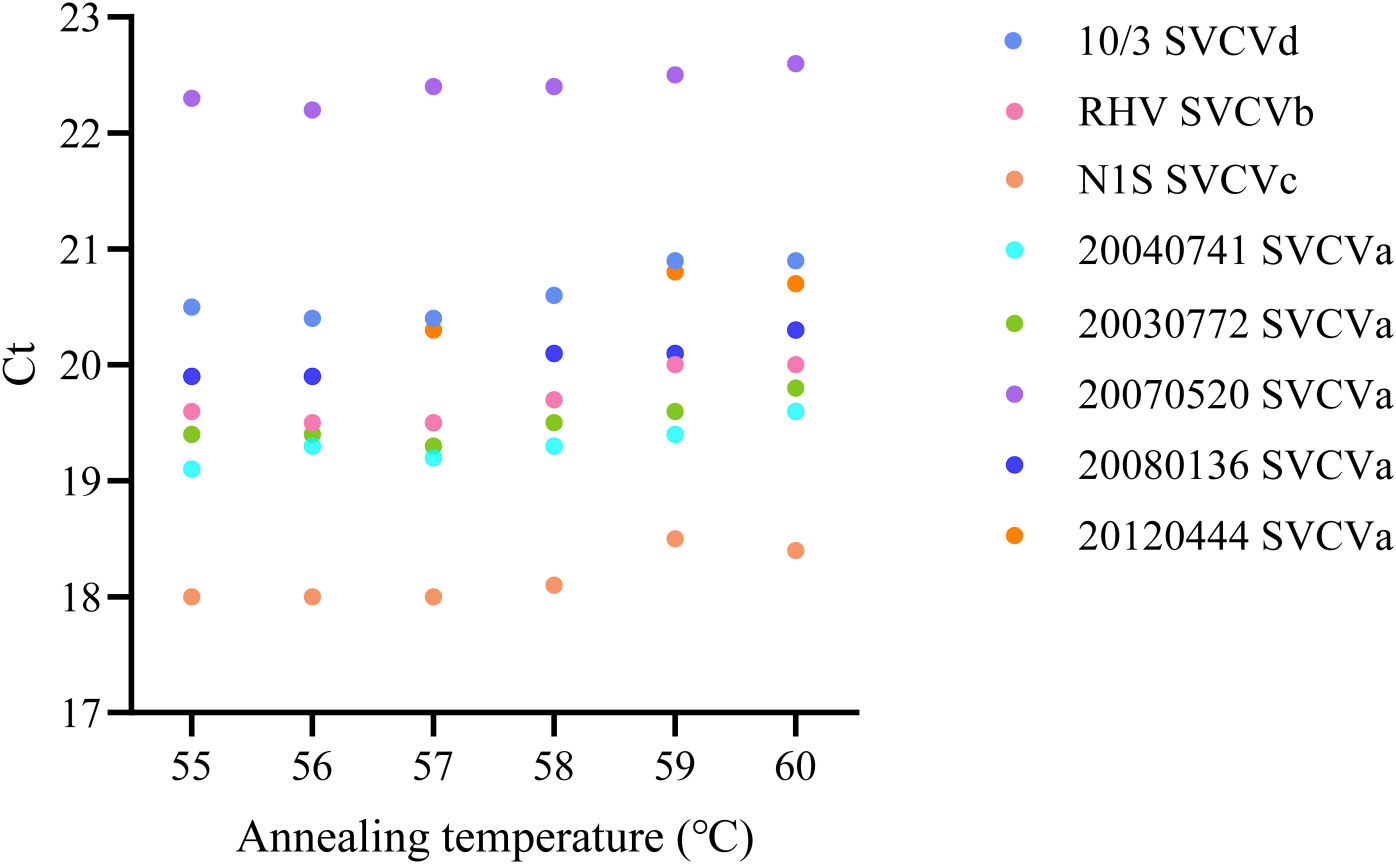
RT-qPCR annealing temperature screening for SVCV a, b, c and d subtypes.

In this study, we optimized the annealing temperature of the RT-qPCR assay using viral RNA extracted from eight representative SVCV strains. The results showed that the minimum Ct values for different strains were mainly distributed between 55 and 57°C. However, the differences compared with 58°C were ≤ 0.2 Ct for most strains, indicating a negligible impact on detection sensitivity (Figure 2). Considering that higher annealing temperatures can reduce non-specific primer binding while still allowing efficient binding to the target sequence, we ultimately selected 58 °C as the optimal annealing temperature for this assay, thereby improving specificity without compromising sensitivity. Furthermore, the primers and probe designed in this study target a highly conserved region of the L gene and successfully detected SVCV isolates covering all four genogroups (SVCV a-d), further demonstrating the broad applicability and high reliability of this method across diverse genotypes.

### 3.2 Analytical specificity and analytical sensitivity

**FIGURE 3:**
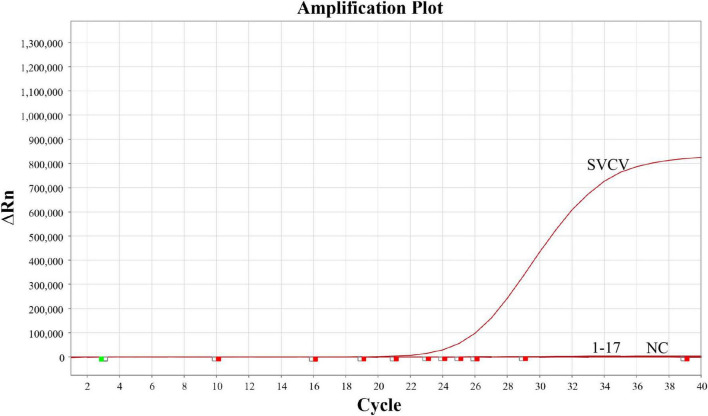
Analytical specificity of the SVCV RT–qPCR assay. Samples 1–17 represent nine viruses: MSRV, LMBV, IPNV, IHNV, GCHV, VHSV, GFHNV, HRV, and KHV; as well as muscle tissue samples from eight fish species: grass carp (*Ctenopharyngodon idella*), crucian carp (*Carassius carassius*), common carp (*Cyprinus carpio*), silver carp (*Hypophthalmichthys molitrix*), bighead carp (*Aristichthys nobilis*), black carp (*Mylopharyngodon piceus*), zebrafish (*Danio rerio*), and mandarin fish (*Siniperca chuatsi*).

The analytical specificity and analytical sensitivity of the developed RT-qPCR assay for SVCV were evaluated. For analytical specificity, exclusivity testing was performed using nine non-target viruse—MSRV, LMBV, IPNV, IHNV, GCHV, VHSV, GFHNV, HRV, and KHV—as well as muscle tissues from eight fish species. No positive signals were detected in any of the non-target viruses or tissue samples (Figure 3), whereas the target SVCV strain was consistently detected. These results indicate that the developed RT-qPCR assay has satisfactory specificity with no observable cross-reactivity.

**FIGURE 4:**
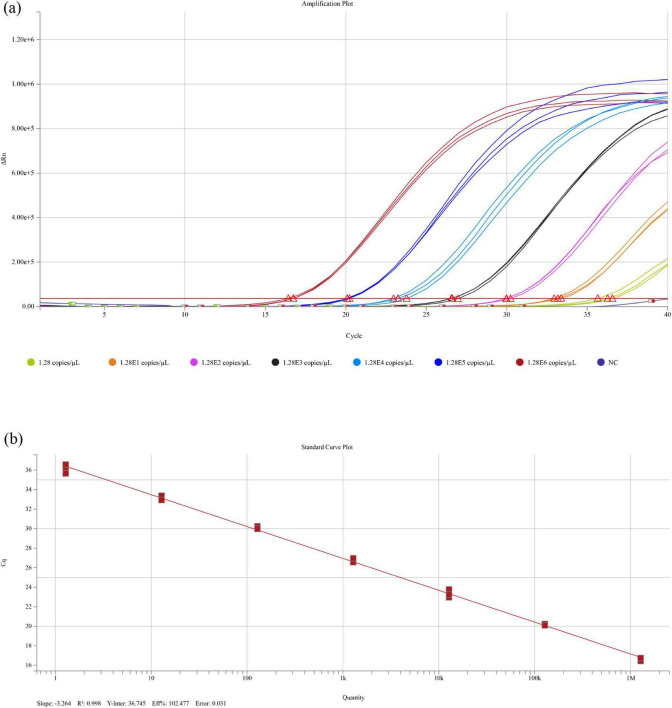
Analytical sensitivity of the RT–qPCR assay. **(a)** Amplification plots of the optimized SVCV RT–qPCR assay with ten-fold serial dilutions of plasmid containing the SVCV L gene. Different colors indicate different plasmid copy numbers, as shown in the legend. NC: negative control. **(b)** Standard curve generated by plotting the Cq values against the log_10_-transformed plasmid copy numbers. The slope, y-intercept, correlation coefficient (R^2^), efficiency, and error of the assay are indicated at the bottom of the plot.

For analytical sensitivity, serial dilutions of the standard plasmid (pUC57-SVCV) were prepared, with three replicates per dilution to determine the limit of detection (LOD). The assay was able to detect positive signals at the lowest concentration of 1.28 copies/μL (Figure 4), indicating a good analytical sensitivity. Based on the standard curve established in this study, Ct = −3.264 × log_10_ (copies)+36.745 (*R*^2^ = 0.998), a theoretical Ct of 36.745 corresponds to one copy of the target template. At this extremely low concentration, stochastic variation caused by sampling volume and sample homogeneity may affect detection consistency. To minimize the risk of false negatives, this value was rounded up, and Ct ≤ 37 was defined as the positivity threshold.

For samples containing ten copies (log_10_ = 1), the calculated Ct value is 33.481. To ensure reliable detection within this low-copy range, even in the presence of minor dilution or pipetting deviations, this value was rounded down, and Ct ≤ 33 was designated as the range of reliable positivity.

Accordingly, Ct values in the range of 33 < Ct ≤ 37 were defined as a suspect zone. Samples within this interval may contain low-copy viral targets and are more susceptible to measurement variability. Therefore, viral isolation and semi-nested RT-PCR are recommended for further confirmation.

### 3.3 Assay repeatability

Based on the repeatability evaluation using three plasmid concentrations, each with three technical replicates, the intra-assay coefficient of variation (CV) for the SVCV L gene ranged from 0.44 to 1.07%, indicating good stability under identical experimental conditions (Table 5). To further verify repeatability for viral nucleic acid detection, the viral RNA of two SVCV strains, 20040741 and 10/3, were assayed at three concentration levels with three technical replicates each. Across all conditions, the triplicate Ct values were highly consistent: standard deviations did not exceed 0.5 and CVs were below 1.5% (Table 6). Inter-assay variability, assessed across three operators, showed CVs ranging from 0.14 to 0.68%. Moreover, for viral RNA from SVCV samples representing different genotypes, detection using the Cefas AR primer set yielded CVs between 0.19 and 2.12%, demonstrating good concordance among operators (Table 7). Overall, the established RT-qPCR assay exhibited excellent repeatability and reproducibility across different concentrations, operators, and experimental runs, and can be reliably used for quantitative detection of SVCV nucleic acid.

**TABLE 5:** Repeatability and inter-assay variability analysis of SVCV RT-qPCR was performed using three concentrations of standard plasmid, with three replicates per concentration.

Plasmid concentration	Intra-assay									Inter-assay	
	Operator 1			Operator 2			Operator 3			Average Ct ± S.D.	CV (%)
	Ct	Average Ct ± S.D.	CV (%)	Ct	Average Ct ± S.D.	CV (%)	Ct	Average Ct ± S.D.	CV (%)		
1.28 E2	30.241	30.069 ± 0.150	0.498%	29.756	30.143 ± 0.284	0.943%	30.369	30.072 ± 0.257	0.856%	30.095 ± 0.042	0.139%
	29.971			30.44			29.94				
	29.994			30.233			29.908				
1.28 E4	23.426	23.365 ± 0.102	0.437%	23.774	23.725 ± 0.223	0.940%	23.736	23.670 ± 0.116	0.490%	23.587 ± 0.159	0.675%
	23.423			23.969			23.738				
	23.246			23.431			23.536				
1.28 E6	16.736	16.636 ± 0.178	1.067%	16.738	16.586 ± 0.135	0.811%	16.646	16.581 ± 0.132	0.799%	16.601 ± 0.030	0.179%
	16.431			16.536			16.669				
	16.741			16.483			16.429				

**TABLE 6:** Repeatability experiment results for SVCV RT-qPCR.

Dilution	Repeat 1 (Ct)	Repeat 2 (Ct)	Repeat 3 (Ct)	Mean ± standard deviation	Coefficient of variation (%)
20040741–10^–2^	24.7	24.2	24.5	24.47 ± 0.25	1.03
20040741–10^–4^	31.2	31.4	31	31.20 ± 0.20	0.64
20040741–10^–6^	37.1	36.6	37.5	37.07 ± 0.45	1.22
10/3–10^–2^	22.8	23	23	22.93 ± 0.12	0.5
10/3–10^–4^	29.9	30.2	29.8	29.97 ± 0.21	0.69
10/3–10^–6^	36	35.9	36.3	36.07 ± 0.21	0.58

**TABLE 7:** Intra-laboratory detection results by different operators at Shenzhen Customs.

Strains	Operator 1	Operator 2	Operator 3	Mean ± standard deviation	CV (%)
RHV	17.3	17.3	17.1	17.23 ± 0.12	0.67
N1S	27.6	27.9	27.6	27.70 ± 0.17	0.63
20040741	15.5	15	14.9	15.13 ± 0.32	2.12
20030772	21.4	21.5	21.5	21.47 ± 0.06	0.27
20070520	31.3	31	31	31.10 ± 0.17	0.56
10/3	30.6	30.5	30.6	30.57 ± 0.06	0.19
20080136	22	22.4	22.2	22.20 ± 0.20	0.9

### 3.4 Diagnostic sensitivity and diagnostic specificity

The RT-qPCR assay established in this study was evaluated using 264 cell-culture isolates. The results were in complete agreement with those obtained using the gold standard method, with no false positives or false negatives observed. These findings indicate that the assay achieved 100% diagnostic sensitivity and 100% diagnostic specificity for cell-culture isolates. When testing tissue samples, 204 specimens previously confirmed by virus isolation and conventional RT-PCR (including gill, spleen, and kidney tissues) were analyzed, of which 7 yielded false-negative results. Notably, all 7 false-negative samples originated from Koi carp infected with the 20100910 strain. Previous studies have indicated that fish infected with this strain were in an asymptomatic (subclinical) state, which may explain the false-negative results (Emmenegger et al., 2022). Therefore, the assay demonstrated a diagnostic sensitivity of 96.6% and a diagnostic specificity of 100% for clinical tissue samples (Table 8).

**TABLE 8:** Evaluation of diagnostic sensitivity (DSe) and specificity (DSp) of the assay based on RT-qPCR.

Sample type	Genotype	Number	Virus isolation	Nested RT-PCR	RT-qPCR	DSe	DSp
Cell-culture isolate	SVCVa	256	(+)256/(−)0	(+)256/(−)0	(+)256/(−)0	100%	100%
	SVCVb	2	(+)2/(−)0	(+)2/(−)0	(+)2/(−)0		
	SVCVc	3	(+)3/(−)0	(+)3/(−)0	(+)3/(−)0		
	SVCVd	3	(+)3/(−)0	(+)3/(−)0	(+)3/(−)0		
Experimental infection	20040741	63	(+)63/(−)0	(+)63/(−)0	(+)63/(−)0	96.6%	100%
	10–3	63	(+)63/(−)0	(+)63/(−)0	(+)63/(−)0		
	20100910	63	(+)63/(−)0	(+)63/(−)0	(+)56/(−)7		
Field samples	None	15	(+)0/(−)15	(+)0/(−)15	(+)0/(−)15		

**FIGURE 5:**
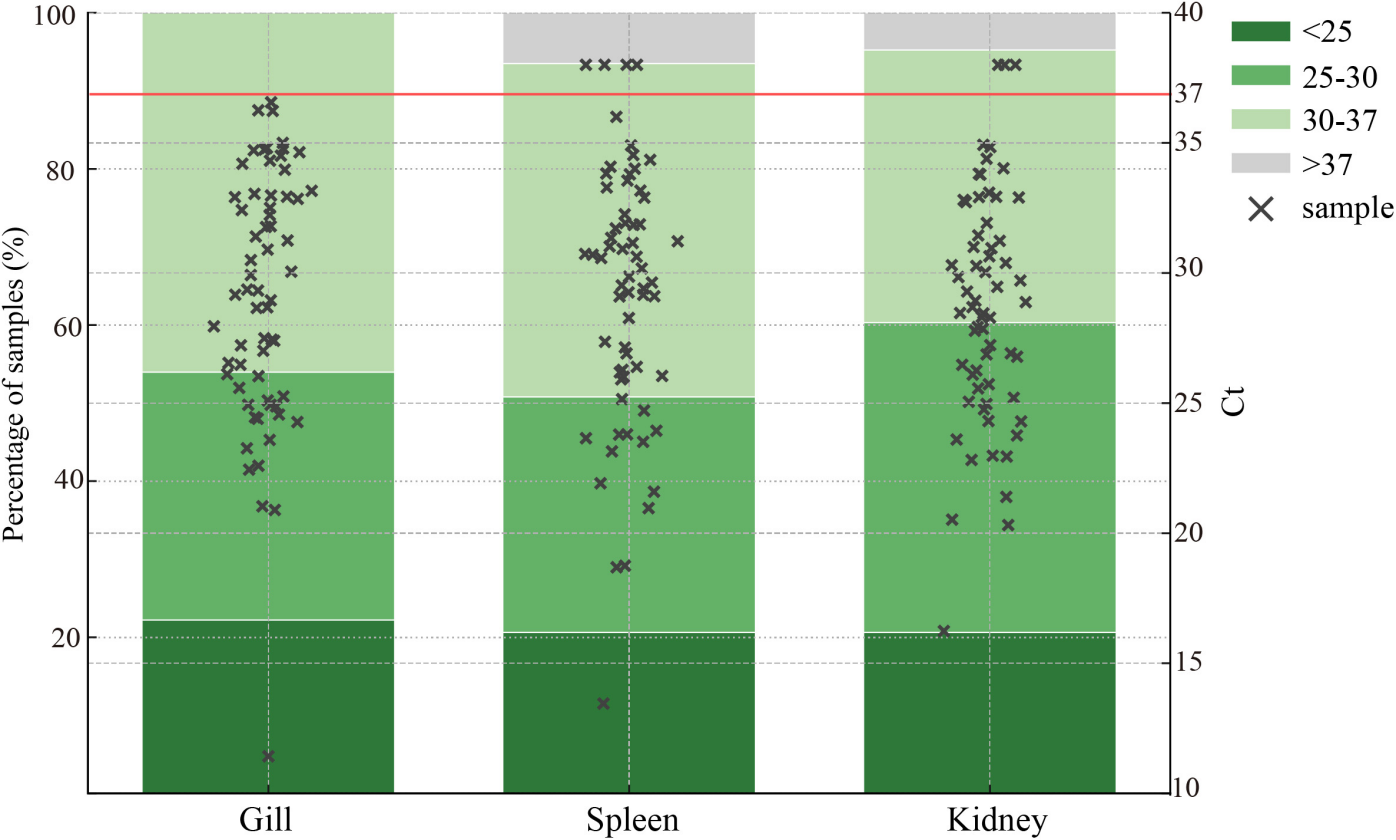
Positive detection rates of RT-qPCR in different tissues. Each tissue type (gill, spleen, and kidney) shows the percentage of samples falling within specific Ct value ranges: <25, 25–30, 30–37, and >37. Individual sample Ct values are indicated by black crosses (×). Samples with Ct ≤ 37 were considered positive. The red line at Ct = 37 represents the positivity threshold.

Further analysis of tissue detection results revealed differences in Ct value distributions among tissue types (Figure 5). The mean Ct values for gill samples were slightly lower than those for spleen and kidney, suggesting higher viral loads in gill tissues. Although several samples exhibited Ct values near the detection limit (approximately 37), resulting in a slight decrease in detection efficiency, most samples remained within the detectable range, and the proportion of undetected samples was low.

In summary, the RT-qPCR assay developed in this study allows efficient, sensitive, and specific detection of cell-culture isolates and tissue samples. However, because the majority of evaluated tissue samples were from experimentally infected or subclinically infected fish, further studies are needed to assess its diagnostic performance in clinically infected fish and under field conditions. Therefore, while the assay provides a practical tool for laboratory detection, its performance in broader surveillance and clinical diagnostics requires additional validation.

### 3.5 Assay reproducibility

The multi-laboratory comparison showed that the RT-qPCR assay established in this study achieved 100% concordance across all known positive and negative blind samples, with no false positives and no false negatives, indicating high accuracy and reliability. Differences among laboratories were most evident for weakly positive samples with Ct values from 31 to 35 (Table 7). LabLYG reported the highest Ct value of 34.51, whereas LabMC and LabZJ reported lower values of 28.66 and 28.65, respectively, suggesting variability in detection sensitivity under low viral load conditions. Results for moderately positive samples with Ct values from 25 to 31 were relatively consistent, with most values clustered between 25 and 30. Strongly positive samples with Ct values from 20 to 25 were the most stable across laboratories and showed minimal variation, indicating that higher viral loads yield more consistent and reliable results. Overall, complete agreement was observed both among laboratories and among different operators within the same laboratory, demonstrating excellent stability and reproducibility across different testing platforms, reagent sources, and personnel.

The observed inter-laboratory differences primarily arose from nucleic acid extraction reagents and workflows, RT-qPCR system composition including premix formulation, enzyme performance and probe stability, and differences in operational practices (Table 9). For example, differences in extraction efficiency and purity between LabQD and LabSH produced an overall shift of about three Ct cycles across strongly positive, moderately positive and weakly positive samples, underscoring the critical impact of RNA extraction on result concordance. Differences between amplification systems, such as those used in LabGB and LabLYG, also had a marked effect on analytical sensitivity (Table 10). For the same specimen, the maximum inter-laboratory difference exceeded five Ct cycles, consistent with order-of-magnitude differences in template copy number. Under ideal amplification efficiency, a difference of about 3.3 cycles corresponds to a tenfold change in copy number, and for some moderately positive samples pairwise differences approached ten cycles, which corresponds to roughly a thousand-fold difference.

**TABLE 9:** Reproducibility different experiment results for SVCV RT-qPCR.

Lab	SVCV strong	SVCV moderate	SVCV weak	Negative 1	Negative 2
LabSZ	POS (25.83)	POS (28.53)	POS (31.47)	Neg	Neg
LabQD	POS (26.27)	POS (30.13)	POS (33.31)	Neg	Neg
LabMC	POS (22.40)	POS (25.19)	POS (28.66)	Neg	Neg
LabZJ	POS (22.18)	POS (25.40)	POS (28.65)	Neg	Neg
LabGB	POS (22.36)	POS (26.00)	POS (29.38)	Neg	Neg
LabGZ	POS (24.14)	POS (28.39)	POS (31.74)	Neg	Neg
LabSH	POS (23.56)	POS (24.57)	POS (27.44)	Neg	Neg
LabXM	POS (23.99)	POS (28.07)	POS (33.09)	Neg	Neg
LabLYG	POS (27.57)	POS (33.34)	POS (34.51)	Neg	Neg

POS, Positive; Neg, not detected.

**TABLE 10:** Information on the reagent kits and reaction protocols used by laboratories.

Laboratory	Sample condition	Nucleic acid extraction kit	Reaction conditions	Real-time RT-PCR Kit
LabSZ	Frozen	Mag MAX-96 Viral Isolation Kit (Thermo Fisher Scientific)	45C° for 10min; 95C° for 10min; 95C° for 15s, 55C° for 45s, 40 cycles	AgPath-ID One-step RT-PCR Kit (ABI)
LabQD	Frozen	Animal Virus DNA/RNA Rapid Extraction Kit 5.0 (TIANLONG)	55°C for 15min; 95°C for 30s; 95° for 10s, 60C° for 34s; 45 cycles	HiScript III U+ One Step qRT-PCR Probe 5 × Master Mix (Vazyme)
LabMC	Frozen	Magnetic Bead-based Ultra-Rapid Viral Nucleic Acid Extraction Kit (Zhongkebio Med Technol)	53°C for 10min; 95°C for 2min; 95°C for 3s, 60°C for 30s, 40 cycles	TaqPath DuraPlex 1-Step RT-qPCR Master Mix (ABI)
LabZJ	Frozen	Mag MAX™-96 Viral Isolation Kit (Thermo Fisher Scientific)	42°C for 30min; 92°C for 3min; 92°C for 10s, 60°C for 30s, 40 cycles	FastKing One Step RT-qPCR Kit (Probe) (TIANGEN Biotech)
LabGB	Frozen	Animal Virus DNA/RNA Rapid Extraction Kit 4.0 (TIANLONG)	42°C for 5min; 95°C for 30s; 95°C for 5s; 55°C for 30s; 40 cycles	Evo-M-MLV One Step RT-qPCR Kit (Accurate Biotechnology (Hunan) Co., Ltd.)
LabGZ	Frozen	MiniBEST Viral RNA/DNA Extraction Kit Ver.5.0 (TAKARA)	42°C for 5min; 95°C for 10s; 95°C for 5s, 60°C for 34s; 40 cycles	TAKARA One Step PrimeScript RT-PCR Kit (AO10560A)
LabSH	Frozen	Magabio Plus Viral RNA Purification Kit (bioflux)	50° for 15 min; 95° for 30 s; 95° for 10 s; 60° for 30 s; 40 cycles	HiScript III U+ One Step qRT-PCR Probe 5 × Master Mix (Vazyme)
LabXM	Frozen	Nucleic Acid Extraction and Purification Reagent (InnoRech)	50°C for 15min; 95°C for 30s; 95°C for 10s, 58°C for 30s; 45 cycles	WinScript Universal One Step RT-qPCR Kit (Cowin Biotech)
LabLYG	Frozen	Animal Virus DNA/RNA Rapid Extraction Kit 4.0 (TIANLONG)	50°C for 30min; 92°C for 3min; 92°C for 10s, 60°C for 30s; 40 cycles	One Step PrimeScript RT-PCR Kit (TAKARA)

In aggregate, the RT-qPCR assay demonstrated robust performance across multiple laboratories, with strong reproducibility and practical utility, and is particularly well suited for samples with moderate to high viral loads. The findings further show that nucleic acid extraction efficiency and the performance of the amplification system are key determinants of assay sensitivity (Table 10).

## 4 Discussion

SVCV is a major pathogen in aquaculture and has caused substantial economic losses worldwide (Ahne, 1978). Genomic divergence exists among different SVCV genotypes, and the virus also exhibits antigenic similarity to related rhabdoviruses, including pike rhabdovirus (Stone et al., 2003; Vicenova et al., 2011). Therefore, conventional immunoassays and RT-PCR may lead to target misclassification (Dixon and Longshaw, 2005). Monoclonal antibody assays have low sensitivity and often fail to detect low viral load infections, whereas polyclonal antibodies may cause cross-reactivity, primarily due to nonspecific binding (Jacobson, 1998; Ashraf et al., 2016). Therefore, molecular diagnostic methods are essential for SVCV detection, among which RT-qPCR is the preferred approach for achieving highly accurate SVCV detection (Koutná et al., 2003; Stone et al., 2003; Liu et al., 2008).

In recent years, RT-qPCR detection methods for SVCV have primarily targeted the G and N genes. Yue et al. (2008) designed a TaqMan assay based on the conserved region of the G gene using then-available limited sequence data. They validated its specificity using five Chinese and UK isolates, reporting a limit of detection of approximately 40 copies/μL. However, due to the heavy reliance on sequences from genogroup Ia, this method exhibited limitations in cross-genogroup detection (Yue et al., 2008). Shao et al. (2016) developed an assay targeting the more conserved and highly expressed N gene, which covered Asian isolates (SVCV-265, SH140501, SH140502, SH140503, and SH140522) and the European representative strain 10/3 (genogroup SVCVd). Its higher sensitivity, specificity, and reliability were demonstrated across multiple cell lines and over 1,100 field samples, significantly improving consistency in detecting various genogroups. The reported limit of detection was 9 copies/μL (Shao et al., 2016). Clouthier et al. (2021b) established two assays: Q1G (targeting G) and Q2N (targeting N). The Q2N assay had a detection limit of 5 copies/μL and consistently detected all four major genogroups (SVCVa-d). In contrast, Q1G occasionally missed some distantly related strains and had a higher detection limit of 12.5 copies/μL (Clouthier et al., 2021b; Clouthier et al., 2021a).

**FIGURE 6:**
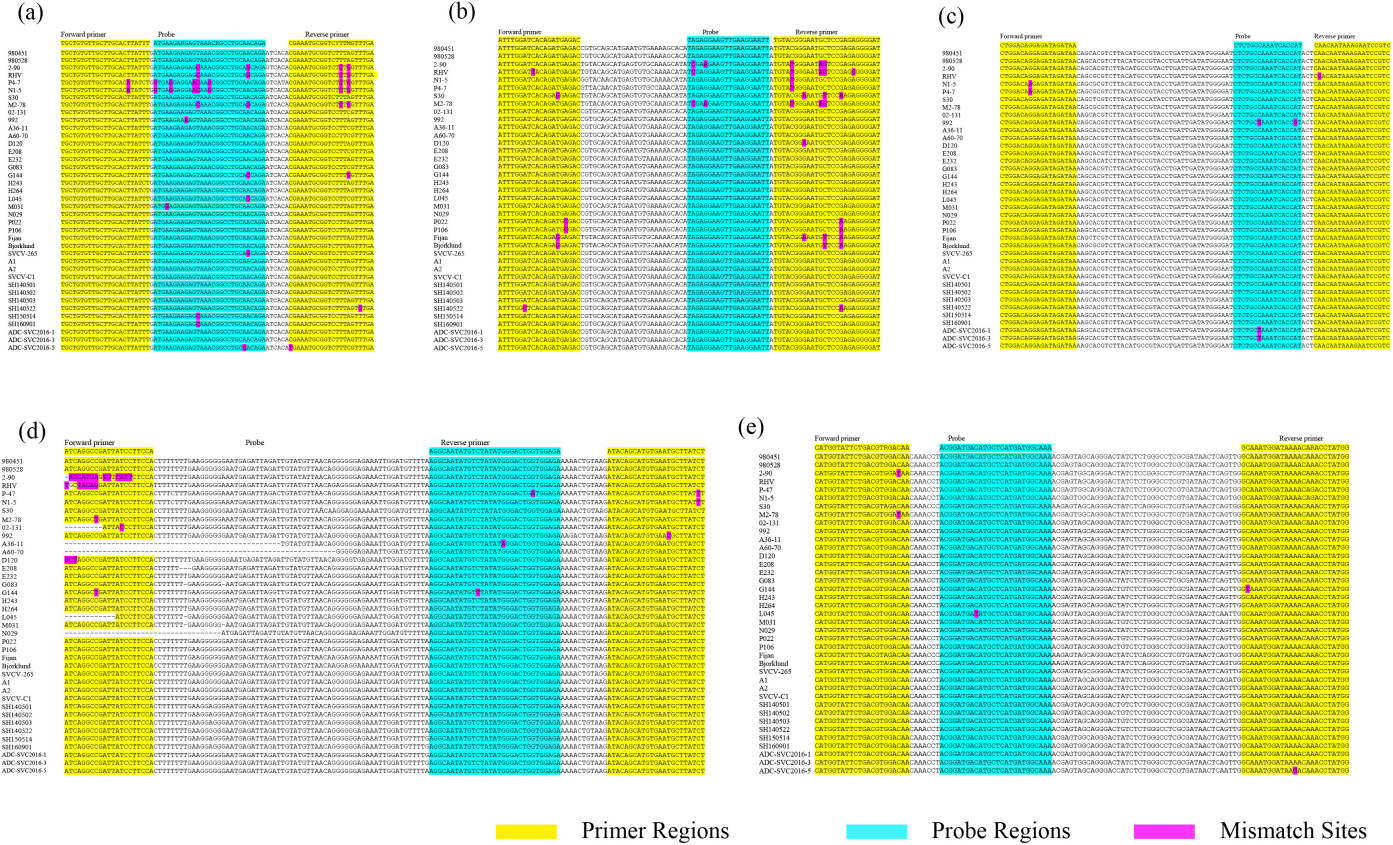
Alignment of the SVCV gene regions from previously published TaqMan RT-qPCR assays **(a–e)**. Primer sequences are highlighted in pink and probe sequences are highlighted in blue. The sequence divergence observed between isolates is highlighted. **(a)** G gene detection method developed by Yue et al.; **(b)** G gene detection method established by Clouthier et al.; **(c)** N gene detection method established by Clouthier et al.; **(d)** N gene detection method 1 established by Shao et al.; **(e)** N gene detection method 2 established by Shao et al.

Further analysis revealed that published assays contain various degrees of primer and probe mismatches, which compromise detection accuracy. For example, the Yue et al. assay has 7 mismatches against genogroup Ic and 4 against Id (Figure 6a); Clouthier et al. also reported one mismatch in the forward primer and three in the reverse primer when aligned with the Fijan strain (Figures 6b,c); and Shao et al. established two RT-qPCR assays that contain several nucleotide mismatches within both the primer and probe regions (Figures 6d,e). These mismatches may reduce detection efficiency, particularly for highly variable strains.

To address this issue, we developed a novel RT-qPCR assay based on the highly conserved L gene—the Cefas AR assay. By incorporating degenerate bases in the primer design, we achieved exact matches against all major genogroups, ensuring full complementarity between primers/probe and target sequences. Compared to existing methods, this system offers improved sensitivity and specificity, making it suitable for global surveillance and rapid screening. Although the assay does not differentiate subgenogroups, it comprehensively covers all four major SVCV genogroups (SVCVa-d), significantly reducing the risk of false negatives due to genetic variation. The assay was validated according to the WOAH Manual of Diagnostic Tests for Aquatic Animals and demonstrated a limit of detection of 1.28 copies/μL, high analytical stability, and no nonspecific amplification when tested against MSRV, LMBV, IPNV, IHNV, GCHV, VHSV, GFHNV, HRV, KHV or cyprinid tissue samples. The diagnostic specificity was confirmed to be 100%.

The RT-qPCR assay established in this study demonstrated excellent sensitivity, specificity, and coverage of major SVCV genotypes, but several limitations should be noted. First, the analytical performance evaluation used the standard plasmid pUC57-SVCV rather than in vitro transcribed RNA, which may not fully replicate the properties of viral RNA. Second, the diagnostic performance was assessed using samples from laboratory infection experiments, which may not entirely reflect natural infection conditions. Although the results indicated a high detection rate in clinical tissue samples, these samples were limited to a few independent trials, and further validation using a larger set of field samples is warranted. Finally, the implementation of the assay under field conditions (Stage 4) has not yet been fully completed, which could be addressed in future studies to achieve full validation.

Overall, despite these limitations, the RT-qPCR assay developed in this study provides a reliable and practical tool for laboratory detection, epidemiological surveillance, and potential field applications of SVCV. These limitations highlight directions for future optimization and expanded use but do not compromise the assay’s effectiveness and applicability under the primary experimental conditions.

## 5 Conclusion

In this study, we developed and validated a RT-qPCR assay (Cefas AR) targeting a conserved region of the SVCV L gene for detection across genogroups SVCVa-d. The assay showed excellent analytical performance (LoD 1.28 copies/μL) with no cross-reactivity to non-target pathogens. Across 264 cell-culture isolates, diagnostic sensitivity and specificity were both 100%, and inter-laboratory repeatability and reproducibility were demonstrated in a nine-laboratory collaborative trial. When applied to tissue samples, the diagnostic sensitivity was 94% and the diagnostic specificity was 100%. For borderline or low viral load samples with 33 < Ct ≤ 37, it is recommended to confirm results using cell culture and RT-PCR. Taken together, these data indicate that the assay meets WOAH validation principles (analytical performance, diagnostic performance, repeatability, reproducibility) and is suitable for rapid screening, routine surveillance and confirmatory diagnosis across genogroups SVCVa-d. The assay can therefore support movement control, outbreak response and trade biosecurity in cyprinid aquaculture.

## Data Availability

The data presented in this study are deposited in the GenBank repository under accession numbers PX727022–PX727045.
