# Correction: Development and partial validation of an RT-qPCR assay for the rapid detection of spring viremia of carp virus (SVCV)

**DOI:** 10.3389/fmicb.2026.1805875

**Published:** 2026-02-24

**Authors:** Frontiers Production Office

**Affiliations:** Frontiers Media SA, Lausanne, Switzerland

**Keywords:** diagnostic assay, fish disease surveillance, RT-qPCR, spring viremia of carp virus, viral detection, WOAH validation

Affiliations 1 and 2 were erroneously ordered in the opposite manner. Accordingly, the correct author and affiliation order appear below:

Peng Zhu^1, 2, 3, †^ Jie Sun^1†^, Lishan Liao^1^, Zhiheng Zuo^4^, Annabel Rice^5^, Shishun Gui^3^, Jiang Wu^1^, Yumin Zhu^1^, Lei Zhang^1^, Hongwei Liu^6^, David Stone^5*^ and Hong Liu^1*^

^1^Animal and Plant Inspection and Quarantine Technology Centre, Shenzhen Customs, Shenzhen, China

^2^College of Life Sciences and Oceanography, Shenzhen University, Shenzhen, China

^3^College of Fisheries and Life Sciences, Shanghai Ocean University, Shanghai, China

^4^College of Life Science and Technology, Jinan University, Guangzhou, China

^5^Center for Environment, Fisheries and Aquaculture Science, Weymouth, United Kingdom

^6^Comprehensive Technical Service Center of Dandong Customs, Dandong, Liaoning, China

There was a mistake in content in **Figure captions 3 and 5** as published. The captions were erroneously cut short.

Figure 3. Analytical specificity of the RT–qPCR Assay for SVCV.

Figure 5. Positive detection rates of RT-qPCR in different tissues (Ct ≤ 37 Considered Positive).

The corrected captions appear below.

Figure 3. Analytical specificity of the SVCV RT–qPCR assay. Samples 1–17 represent nine viruses: MSRV, LMBV, IPNV, IHNV, GCHV, VHSV, GFHNV, HRV, and KHV; as well as muscle tissue samples from eight fish species: grass carp (*Ctenopharyngodon idella*), crucian carp (*Carassius carassius*), common carp (*Cyprinus carpio*), silver carp (*Hypophthalmichthys molitrix*), bighead carp (*Aristichthys nobilis*), black carp (*Mylopharyngodon piceus*), zebrafish (*Danio rerio*), and mandarin fish (*Siniperca chuatsi*).

Figure 5. Positive detection rates of RT-qPCR in different tissues. Each tissue type (gill, spleen, and kidney) shows the percentage of samples falling within specific Ct value ranges: < 25, 25–30, 30–37, and >37. Individual sample Ct values are indicated by black crosses ( × ). Samples with Ct ≤ 37 were considered positive. The red line at Ct = 37 represents the positivity threshold.

There was a mistake in Table 8 as published. In the published version, the value in the “Number” column for the field samples was left blank. This blank entry has been corrected to **15**. The corrected table appears below.

**Table 8:** Evaluation of diagnostic sensitivity (DSe) and specificity (DSp) of the assay based on RT-qPCR.

**Sample type**	**Genotype**	**Number**	**Virus isolation**	**Nested RT-PCR**	**RT-qPCR**	**DSe**	**DSp**
Cell-culture isolate	SVCVa	256	(+)256/(–)0	(+)256/(–)0	(+)256/(–)0	100%	100%
	SVCVb	2	(+)2/(–)0	(+)2/(–)0	(+)2/(–)0		
	SVCVc	3	(+)3/(–)0	(+)3/(–)0	(+)3/(–)0		
	SVCVd	3	(+)3/(–)0	(+)3/(–)0	(+)3/(–)0		
Experimental infection	20040741	63	(+)63/(–)0	(+)63/(–)0	(+)63/(–)0	96.6%	100%
	10-3	63	(+)63/(–)0	(+)63/(–)0	(+)63/(–)0		
	20100910	63	(+)63/(–)0	(+)63/(–)0	(+)56/(–)7		
Field samples	None	15	(+)0/(–)15	(+)0/(–)15	(+)0/(–)15		

The original version of this article has been updated.

